# Role of RUNX2 in Breast Carcinogenesis

**DOI:** 10.3390/ijms160920969

**Published:** 2015-09-02

**Authors:** Daniel Wysokinski, Janusz Blasiak, Elzbieta Pawlowska

**Affiliations:** 1 Department of Molecular Genetics, University of Lodz, Pomorska 141/143, 90-236 Lodz, Poland; E-Mail: dwysokinski@gmail.com; 2 Department of Orthodontics, Medical University of Lodz, Pomorska 251, 92-216 Lodz, Poland; E-Mail: elzbieta.pawlowska@umed.lodz.pl

**Keywords:** RUNX2, estrogen, breast cancer, DNA damage response, cell cycle

## Abstract

RUNX2 is a transcription factor playing the major role in osteogenesis, but it can be involved in DNA damage response, which is crucial for cancer transformation. RUNX2 can interact with cell cycle regulators: cyclin-dependent kinases, pRB and p21Cip1 proteins, as well as the master regulator of the cell cycle, the p53 tumor suppressor. RUNX2 is involved in many signaling pathways, including those important for estrogen signaling, which, in turn, are significant for breast carcinogenesis. RUNX2 can promote breast cancer development through Wnt and Tgfβ signaling pathways, especially in estrogen receptor (ER)-negative cases. ERα interacts directly with RUNX2 and regulates its activity. Moreover, the *ER*α gene has a RUNX2 binding site within its promoter. RUNX2 stimulates the expression of aromatase, an estrogen producing enzyme, increasing the level of estrogens, which in turn stimulate cell proliferation and replication errors, which can be turned into carcinogenic mutations. Exploring the role of RUNX2 in the pathogenesis of breast cancer can lead to revealing new therapeutic targets.

## 1. Molecular Aspects of Breast Cancer

### 1.1. Hormone-Dependent Cancers

Some cancers respond specifically to hormonal signals, expressing high level of hormone receptors and they are called hormone-dependent cancers. Furthermore, many tumors are capable of producing hormones, mimicking an endocrine tissue. Tumors dependent on estrogens are represented mainly by breast, endometrium, ovary and prostate cancers [1]. A strong association with estrogen, with 2–4 relative risk increase for high estrogen levels, was reported for endometrial cancer, which is the most common gynecological cancer with increasing incidences in developed countries [2]. Endometrium belongs to the main targets of estrogen control in women [3]. At least one subtype of endometrial cancer (Type-I endometrioid) is proved to be estrogen-dependent, although currently both types I and II, are thought to be linked with estrogen [4]. A weaker association and limited epidemiological data are available on estrogen and ovarian cancer, which has the highest mortality among gynaecological cancers [1,5]. Another group of estrogen-related cancers are endocrine gland cancers, originating from adrenocortical, pancreatic, prostate and thyroid tissues. Thyroid cancer is three to five times more frequent among women than men, and even more common in females in reproductive age [6]. Contraceptive usage and hormone replacement therapy (HRT) result in elevated risk of thyroid and pancreatic cancers [6]. Male-specific prostate cancer has been also included in estrogen-dependent cancers group. It affect individuals of advanced age, when the production of testosterone declined, while estrogen levels remain stable or even rise. It seems that high estrogen/testosterone ratio is a key factor for prostate cancer development [7]. It is the second most common cancer in males, and its incidence between populations varies greatly worldwide [8].

### 1.2. Role of Estrogen in Breast Carcinogenesis

The mortality due to breast cancer has decreased by 25% during past two decades, but it is still the second most common cancer and the most common cancer among women in the world [9,10]. Breast cancer is heterogeneous, with multiple distinct subtypes characterized by different patterns of gene expression, and different a prognosis in each subtype [11]. The link between breast cancer and hormonal state has been known for more than 100 years, when it was found that the removal of ovaries was beneficial for women with breast cancer [12]. The most consistent data describe the link between estrogen levels and breast cancer in postmenopausal women, indicating a significant risk increase associated with high circulating estrogen levels [1]. Supporting data come from investigations of the effect of HRT on breast cancer risk, indicating a strong positive association [13].

Estrogens are synthesized in both females and males, but their levels are significantly higher in pre-menopausal women [14,15]. 17-β-estradiol (E2) is the most active form of estrogen. It is synthesized primarily in the ovarian granulosa cells, but also in Leydig cells in the testis, adipose, and nervous cells [16]. The level of E2 estrogen fluctuates in pre-menopausal females, depending on menstrual cycle phase, and its level drops significantly in menopause [6]. There are also two metabolites of E2, estrone (E1) and estriol (E3), displaying less activity than E2. Aromatase is a central enzyme of estrogen synthesis. It catalyzes the conversion of androgens—androstenedion and testosterone to estradiol and estrone, respectively [17]. Aromatase is a member of the cytochrome P450 superfamily, encoded by the *CYP19* gene. It is expressed in many tissues, including gonads, brain, blood vessels, liver, bone, skin, adipose, and endometrium [18]. Estradiol is a key regulator of fertility in both men and women. It has been identified as a hormone regulating the growth, differentiation, and reproductive processes in females. Its role in the development and function of breast, ovary, and uterus in females, and prostate and testes in males, is well documented [14,19]. Currently, it is known that estrogens regulate a broad variety of physiological processes, including differentiation in many tissues/organs, such as brain, liver or cardiovascular, and skeletal system [14,19]. It also plays a role in inflammatory response, the gastrointestinal tract, and regulation of cognitive and motor functions [14,19].

### 1.3. Estrogen Receptors in Breast Cancer

Two canonical estrogen receptors (ERs) have been identified; ERα and ERβ, which are involved in estrogen sensing and signal transduction in estrogen-dependent pathways. ERα/β belongs to the family of steroid/thyroid nuclear receptors [20]. The central mechanism of estrogen action is the binding of activated ER receptors to estrogen response elements (ERE), although it is now known that estrogens may also act without direct interaction with target genomic sequences [20]. Therefore, there are two distinct, independent pathways of estrogen action within the cell. First, called the “genomic pathway”, which is a canonical way of action through estrogen response elements within genes, and, second, the “non-genomic pathway”. Both pathways are ligand-dependent, but estrogen receptors can also act in a ligand-independent manner. It was shown that ERs could also be activated through alternative signaling pathways triggering phosphorylation events on ERs [21]. Several genes may be regulated by the ER mechanism despite the absence of ERE elements. Such regulation is possible through interaction of active ER with other DNA-bound transcription factors, such as AP-1, SP-1, FOX, oct, NF-κB or GATA3 [6,14,22]. The non-genomic pathway is rapid and depends on ERs anchored in cell membrane or other transmembrane estrogen-binding proteins. Membrane ER activation leads to a series of events, such as calcium or NO signalization and activation of multiple kinases [6,23]. The third mechanism of estrogen action is ligand-independent, and includes other signaling pathways to activate ER. Both ERs can be phosphorylated by MAPK and PI3K kinases [16]. They can also be phosphorylated by ERK and Akt kinases, activated by epidermal growth factor receptor (EGFR) or insulin-like growth factor receptor (IGFR) [24].

Since estrogen controls the growth of several tissues, such as mammary glands and endometrium, it has also the potential to trigger uncontrolled cell proliferation within these tissues. In fact, it is well known that estrogen has a role in breast and endometrium tumor growth [25]. The best known of these is the receptor-dependent modulatory role of estrogen on tumor cell proliferation. In brief, ERα promotes the growth of breast tumors, while ERβ has an opposite effect, inhibiting growth of breast cancer cells [26]. Identification of downstream estrogen-responsive genes seems to be crucial for understanding estrogen’s role in carcinogenesis, but they create a very complex system of interactions, and it seems that there are multiple pathways through which estrogen can act. Additionally, estrogen action on the genomic level, and its role in carcinogenesis are also considered in the physico-chemical background. A high level of both naturally occurring and synthetic estrogens are known to cause adverse consequences, including immunotoxic and carcinogenic effects [27]. Estrogen is linked to enhanced proliferation, decreased apoptosis, and DNA damage in breast cancer [1]. Several experiments on animals have demonstrated that estradiol administration increased the risk of breast cancer, while anti-estrogen agents had an opposite effect [28,29]. Moreover, bilateral oophorectomy (ovary removal) reduced the risk of breast cancer incidence by 75% [30]. A significant increase of breast cancer risk was also linked with high free-plasma estrogen levels [30,31]. Application of tamoxifen, a selective estrogen-receptor modulator (SERM), which blocks estrogen receptors from estrogen binding, reduced breast cancer risk by more than 50%. A similar protective effect displayed inhibitors of aromatase, preventing estrogen conversion from its precursors [31,32]. An emerging body of evidence suggests that the RUNX2 transcription factor, a key regulator of osteogenesis, can play a role in breast carcinogenesis, particularly in proliferation and metastasis.

## 2. RUNX2—A Transcription Factor and the Bone Growth Master Regulator

### 2.1. The Structure of RUNX2 Gene and Protein

RUNX2 is a transcription factor belonging to the RUNX family, characterized by the runt domain [33]. While primitive metazoans have a single RUNX protein, higher organisms have multiple RUNX factors, with three RUNXs in humans, designated RUNX1–3 [34]. The runt domain is located in the N-terminal part of each RUNX factor and there is ~90% homology among them [34]. The *RUNX* genes encode the α subunit of polyomavirus enhancer-binding protein 2/core binding factor (PEBP2/CBF) transcription factors [35], a heterodimeric proteins with DNA-binding and non-DNA-binding subunits. The runt domain is responsible for both, α and β subunit heterodimerization, and interaction with DNA [36,37]. CBFβ, in turn, contains the consensus PyGPyGGTPy sequence and acts through increasing the α subunit-DNA binding affinity and its stabilization, thus, protecting it from proteolytic degradation [34,38]. Each RUNX member also contains a transactivation and an inhibitory domain (ID), both located at the C-terminal part, PPxY (PY) motif enabling RUNX to interact with peptides containing WW domain, and VWRPY motif interacting with the WD domain of Groucho/TLE family transcription co-repressors [34,39]. The RUNX proteins also contain nuclear localization signal (NLS) inside the runt domain and the matrix-targeting signal required for nuclear transactivation [39]. Each RUNX protein is produced in two major isoforms due to two alternative promoters, distal P1 and proximal P2 [40]. Isoforms differ in length and their N-terminal sequences. Type I isoform has MRIPV motif on its N-terminus, while longer type II isoform has MASN/DS motif instead [40]. Both RUNX transcripts undergo additional alternative splicing, generating protein products that are different in structure and specificity [39,41]. They also contain dispersed RUNX-binding sites, probably enabling feedback regulation between RUNX family members, although this mechanism is not fully understood [42].

### 2.2. RUNX2 in Physiology

RUNX2 plays a pivotal role in skeletal development. Haploinsufficiency of the *RUNX2* gene leads to severe skeletal disease cleidocranial dysplasia (CCD), whereas its total inactivation results in complete lack of ossification, as shown on experimental animals [43,44]. RUNX2 transactivates expression of several bone matrix protein encoding genes, including type I collagen, osteoponin, osteocalcin, Col1a1, Col1a2, bone sialoprotein (BSP), and fibronectin [44,45]. Another target for RUNX2 is the Indian hedgehog (Ihh) transcription factor, which is a regulator of chondrogenesis [46]. The type II isoform of RUNX2 plays a major role in osteoblastogenesis, since it is expressed ubiquitously in osteoblasts [47], while type I isoform can play a role in other mesenchymal tissues [45]. The expression of RUNX2 was also found in numerous other tissues and organs, including ovary, testis, and brain, suggesting that this factor could have multiple functions in many organs and systems [39,48].

RUNX2 action in the regulation of gene expression is linked with its cooperation with histone-modifying enzymes. It was shown that RUNX2 interacted with histone deacetylase 6 (HDAC6) in osteoblasts through its C-terminal domain [49]. Although HDAC3 does not directly associate with RUNX2, it binds to the osteocalcin gene promoter, containing a binding site for RUNX2, thus disabling RUNX2 binding to, and activation of, the osteocalcin gene [50]. Moreover, RUNX2 was shown to interact with TLE co-repressors, human homologs of the *Drosophila* Groucho proteins, which are common co-repressors of many genes [51].

### 2.3. Multipathway Regulation of RUNX2

**Figure 1:**
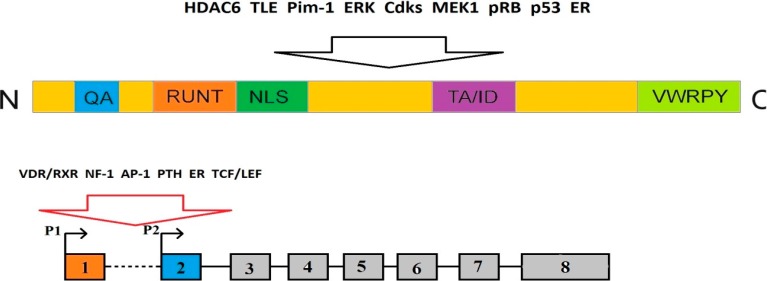
The structure of the RUNX2 protein (**top**) and gene (**bottom**). HDAC6, TLE, Pim-1, ERK, Cdks, MEK1, pRB, p53 and ER denote proteins interacting with RUNX2. VDR/RXR, NF-1, AP-1, parathyroid hormone (PTH), estrogen receptor (ER) and TCF/LEF (T-cell factor/lymphoid enhancer factor) are factors interacting with the *RUNX2* gene promoter. NLS: nuclear localization signal peptide; RUNT: the runt domain; P1 and P2: alternative promoters, numbers in boxes denote exons; QA: polyglutamate and alanine motif; TA/ID: transactivation/inhibitory domain; VWRPY: C-terminal repression motif; VDR/RXR: vitamin D receptor/retinoic acid X receptor.

The intracellular level of RUNX2 and its activity dynamically change depending on cell type, physiological state and external stimuli. It is regulated by a number of post-translational modifications, such as phosphorylation, methylation, acetylation, and ubiquitination [40]. RUNX2 is a target for several kinases, including Pim-1, ERK, or cycline-dependent kinases. The *RUNX2* gene has a regulatory region with multiple binding sites for transcription regulation. It has the vitamin D response element (VDRE), which binds VDR/RXR (vitamin D receptor/retinoic acid X receptor) dimer and is responsible for the suppressive action of 1,25-dihydroxycholecalciferol (1,25-D3) [42]. The *RUNX2* promoter also contains conserved region within the P1 site (−415 to −375), which binds NF-1 and AP1 transcription factors, and functions as tissue-specific enhancer in osteoblasts [52]. RUNX2 is regulated by endocrine signals—it responds to androgens, parathyroid hormone (PTH), and parathyroid-related peptides (PTHrPs) [50]. It was shown that both ERK and MEK1, kinases upstream of ERK, were capable of phosphorylation and, thus, activation of RUNX2 [53]. PI3K-Act positively regulates RUNX2 activity without its phosphorylation, likely through phosphorylation or dephosphorylation of RUNX2 binding partners, thus influencing the ability of such complexes to bind to specific DNA sites [54]. RUNX2 can be also regulated by Wnt signaling pathway. RUNX2 expression may be enhanced by Wnt-dependent TCF/LEF transcription factors directly binding to the *RUNX2* promoter [55,56]. In contrast, cAMP signaling, which is responsible for PTH signals in osteoblasts, decreased RUNX2 level through ubiquitin/proteasome-dependent mechanism [57]. Histone deacetylases can cooperate with RUNX2 in gene regulation, but they also play a role in the regulation of RUNX2 itself. HDACs 3, 4, and 5 are known to suppress RUNX2 activity [51,58,59] (Figure 1).

## 3. Involvement of RUNX2 in DNA Damage Response and Cancer Transformation

### 3.1. The Cellular DNA Damage Response

Because transfer of genetic information requires high fidelity, DNA damage can block the cell cycle, preventing replication of unstable genomes and providing time for DNA damage repair. When the extent of DNA damage exceeds the repair capacity of the cell, it can either tolerate it or activate a programmed death pathway, usually apoptosis, which can protect the cell against cancer transformation. These and some other cellular mechanisms against DNA damage are collectively called the DNA damage response (DDR).

DNA double-strand breaks (DSBs) belong to the most serious DNA damages and, if non-repaired or misrepaired, can lead to cancer transformation or death. DDR starts with damage sensing mechanisms, scanning chromatin for DNA breaks and/or other alterations [60]. The main sensors for DNA damage are MRN (Mre11, Rad50, Nbs1) complex for DSBs and RPA (Replication protein 1) for accumulating single-stranded DNA (ssDNA) in the cell [61]. When DNA damage occurs, signaling pathways are activated to transduce signals to several downstream effectors. Central in initial activation of DDR is ATM protein kinase, once activated it phosphorylates several downstream signaling factors [62]. Other important signaling kinases are ATR and DNA-PK [63]. MRN complex acts through recruitment and activation of ATM kinase, while RPA recruits primarily ATR kinase [60]. Both, ATM and ATR have partially overlapping substrate specificity, preferentially phosphorylating SQ/TQ motifs, which are identified in over 700 proteins [60]. The crucial point in DDR is activation and stabilization of the p53 protein by ATM. Other effectors downstream of ATM are Chk1 and Chk2 [60]. Cell cycle arrest, a major event in DDR, begins with cyclin-dependent kinases (CDKs) inhibition through their phosphorylation on Tyr15 [64,65]. DNA repair plays an important role in DDR. Two major pathways repairing DSBs are homologous recombination repair (HRR) and non-homologous end joining (NHEJ) [66].

### 3.2. Involvement of RUNX2 in DDR

Some research suggests that RUNX2 may be considered as an important factor of the DDR system (reviewed in [67]). The level of RUNX2 in the cell undergoes cell phase-dependent fluctuations, but has different patterns depending on the cell type. In proliferating endothelial cells, it reaches the minimum in the G1 phase and maximum in the G2 or at G2/M transition [68,69], while, in osteoblasts, it reaches a maximum peak in the early G1 phase and the lowest level in the S and M phases [70]. The mechanism underlying these associations is still unknown and some reports are inconsistent. It was shown that RUNX2 overexpression in MC3T3-E1 cells prevented it from G1 to S progression [70]. Moreover, it was shown that the pool of RUNX2 at level alternating with cell cycle phases represented exclusively a type I isoform of RUNX2 [70]. It is known that RUNX2 interacts with a number of cell cycle regulators. It plays a role in the repression of p21Cip1 cyclin-dependent kinase inhibitor, thus promoting cell cycle [71]. Additionally, the activity of RUNX2 is under control of cell cycle regulators. RUNX2 is phosphorylated by Cdk4/cyclin D1, Cdk2/cyclin A and once phosphorylated, it undergoes degradation [34]. However, RUNX2 phosphorylation by Cdk1/cyclin B enhances its binding affinity to DNA [72]. During mitosis, RUNX2 remains associated with specific loci on chromosomes, probably taking part in mitosis progression control [38].

### 3.3. Role of RUNX2 in Cancer

RUNX2 associates also with the major cell cycle negative regulator pRB (retinoblastoma protein) and suppresses its activity, enabling progression of the cell cycle [73]. Since RUNX2 negatively regulates the cell cycle inhibitor, pRB, thus promoting the cell cycle, it may be considered as a potential oncogene. In fact, overexpression of RUNX2 led to the promotion of cell cycle under signals from inhibitors of proliferation [73]. It is now believed that RUNX2 plays a role in cell cycle promotion in various cancers. It is especially seen in leukemias and lymphomas—malignancies of hematopoietic origin [74]. High RUNX2 expression correlates with bad prognosis in BCR-ABL1-positive acute lymphoblastic leukemia (ALL) patients [75]. Introduction of the RUNX2 inhibitor, dexamethasone, upregulates BIM (Bcl-2-interacting mediator of cell death) proapoptotic factor in ALL therapy [76]. However, downregulation of *RUNX2* by shRNA decreased BIM activity and inhibited apoptosis in ALL cells [76]. It was also reported that imatinib, a tyrosine kinase inhibitor used for BCR/ABL1-positive cancers treatment, upregulated RUNX2 expression in human mesenchymal stem cells (hMSCs) at early differentiation stages [77]. RUNX2 is also elevated in many other cancers, such as breast and prostate cancers, gliomas, and it was also correlated with bone metastasis [71,78,79].

**Figure 2:**
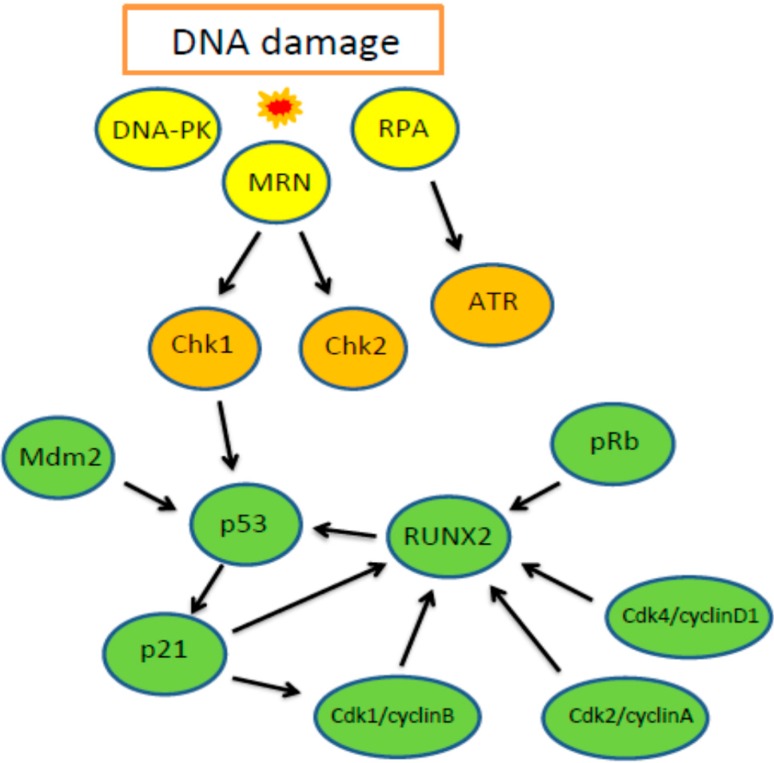
Signaling cascade following DNA damage and important role of RUNX2. The first step is DNA damage sensing through sensor proteins (yellow) to a downstream signaling effectors (orange) to effectors coordinating DNA damage response and determining cell fate (green).

Recent data show that RUNX2 interacts directly with the p53 tumor suppressor and can act in the opposite way as p53. RUNX2 interacts with p53 under genotoxic stress, and such a complex is stable after its recruitment to p53-responsive sites in effector genes [80,81]. Overexpression of RUNX2 led to downregulation of many genes downstream of p53. Moreover, downregulation of RUNX2 also led to elevation of these factors in DNA damage conditions [80,81]. The p21Cip1 cell cycle regulator can be suppressed by the p53/RUNX2 complex [82]. RUNX2 also downregulates the target of p53—the *miRNA-34* gene, which controls cell proliferation [83]. Recent data show that some DDR-related factors can cooperate with RUNX2 in the differentiation process. Additionally, p53 was shown to play a role in bone metabolism. Deficiency of p53 led to enhanced proliferation and differentiation of osteoblasts [84]. Elevated activity of p53 in osteoblasts upregulated RUNX2, leading to the inhibition of osteoblasts differentiation, but without affecting apoptosis [70,85] (Figure 2). RUNX2 appears to be involved in the pathogenesis of several cancer types and it seems that its role in breast cancer is complex and can be strongly bound to estrogen signaling. Both, RUNX2 and estrogen pathways may cooperate at many points.

## 4. Role of RUNX2 in Breast Cancer

### 4.1. Cooperative Action of RUNX2 and Estrogen Signaling in Bone Homeostasis

**Figure 3:**
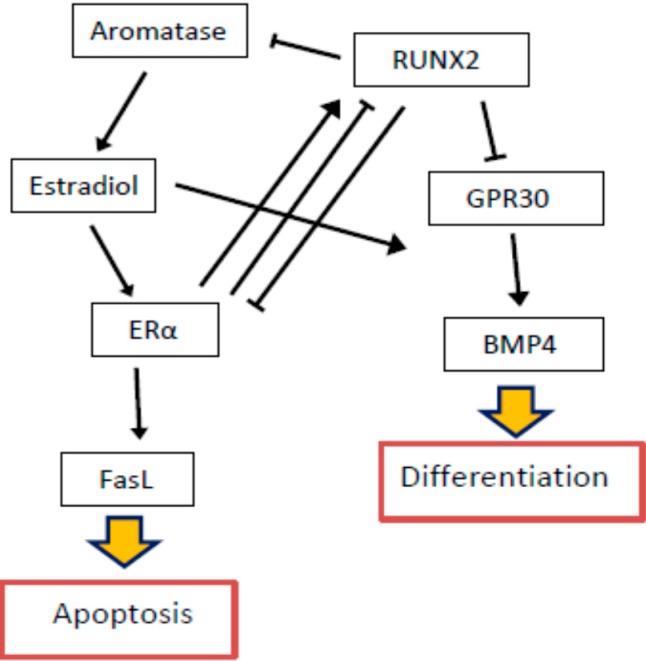
RUNX2 and estrogen signaling pathways in the control of cellular apoptosis and differentiation. Sharp arrows indicate positive, while blunt arrows—negative regulation. FasL: fas ligand; GPR30: G protein-coupled estrogen receptor; BMP-4: bone morphogenetic protein 4.

Some results suggest a functional interaction between RUNX2 and estrogen in skeletal tissues. Estrogen is essential for bone development in both males and females [86]. Estrogen deficiency may lead to osteopenia and osteoporosis, often observed in postmenopausal women [87]. Mutations in genes involved in the estrogen pathway lead to a disrupted bone homeostasis [88]. In osteoblasts, estrogen mediates activation of Fas ligand (FasL) through ERα, inducing their apoptosis [89]. Estrogen can also upregulate osteoprotegerin expression and, thus, inhibit osteoclasts formation [90]. Aromatase, the estrogen-producing enzyme, is expressed in bone cells and its gene contains a bone-specific promoter, producing isoforms with two additional exons on 5′-end of transcript [44,91]. In pathological conditions associated with elevated osteogenesis rate, aromatase is also upregulated [44]. Many factors show a stimulatory effect on aromatase expression. It was shown that RUNX2 regulator, dexamethasone, stimulates both aromatase activity and its expression level in osteoblasts [92]. In the presence of dexamethasone, 1,25-dihydroxyvitamin D3, another RUNX2 regulator, enhances aromatase activity and its expression [93]. Recent data show that RUNX2 in osteoblasts can also induce aromatase gene expression through a direct interaction with its promoter [94]. It suggests that local estrogen production in bone tissue is under the control of RUNX2 factor, and the aromatase gene is a downstream target for RUNX2. Moreover, it was recently shown that RUNX2-dependent local estrogen production in bone is controlled by a positive and negative feedback loop. ERα interacts directly with RUNX2 and regulates its activity depending on the presence of estrogen [94,95]. Moreover, the distal promoter (the F promoter) of the *ER*α gene, located at −117 to 140, contains three potential binding sites for RUNX2 [96]. It was shown that an additional RUNX2 binding site acted as a transcriptional repressor. Deletion at this site in SaOS-2 and hOB cell lines abolished the recruitment of RUNX2 to the regulatory site in *ER*α-encoding gene [97]. Moreover, siRNA-mediated downregulation of RUNX2 in osteoblasts led to upregulation of ERα transcription and activity [97]. Another study demonstrated that estrogen receptors ERα and ERRα (estrogen related receptor α), both, regulated RUNX2 type I isoform transcription and ERRα regulation may be positive or negative, depending on other co-regulators action [98]. Additionally, another estrogen receptor, GPR30, was shown to be controlled through RUNX2 and participated in the control of osteoblasts proliferation [99,100]. In MC3T3-E1 osteoblasts, estradiol has been shown to induce proliferation of early osteoblast progenitors [101]. It enhanced BMP-4 factor, followed by Smad1/5/8 signaling pathway activation, which resulted in enhanced expression of differentiation markers, including alkaline phosphatase [101]. Estrogen is considered as an essential regulator of osteoblasts differentiation and directly cooperates with RUNX2 in the process of differentiation (Figure 3).

### 4.2. Interactions of RUNX2 and Estrogen in Breast Cancer

#### 4.2.1. Estrogen and Estrogen Receptors

The role of RUNX2 in breast cancer may be related to its role in normal mammary gland development. The functioning of RUNX2 begins in mammary stem cells, which generate progenitors, developing into luminal and basal mammary lineages [102]. It was shown that RUNX2 was one of the most important transcriptional regulators in mammary glands development [103]. Estradiol and RUNX2 were reported to have an opposite effect on breast cancer metastatic cells. While estrogen is known to induce breast tumor progression *in situ*, it also displays an anti-metastatic action, acting in an opposite way to RUNX2, promoting breast cancer cells invasion *in vitro* [104]. The connection between both opposite actions seems to be the SNAi2 transcription factor, which can be stimulated by RUNX2 and inhibited by estradiol [105,106]. Overexpression of RUNX2 in MCF7 breast cancer cell line induced epithelial to mesenchymal transition (EMT), with dependence on Wnt and Tgfβ signaling pathways [104]. The pro-metastatic properties of RUNX2 can result from its physiological roles, since it is a key regulator of cellular differentiation, and motility and plays an important role in bone development and turnover, including its vascular invasion. RUNX2 regulates the expression of bone sialoprotein, osteoponin, vascular endothelial growth factor (*VEGF*), and collagenase-3 [103]. All these genes participate in physiological functions of RUNX2 under normal conditions and in the metastatic activity of tumors. This was confirmed in a *RUNX2* downregulation experiment, showing a decreased activity of these genes and a diminished activity of osteolysis [107]. However, some data of RUNX2 expression in breast cancer are inconsistent. It is noteworthy that, in one study, RUNX2 elevated expression was observed in only a subset of breast cancers [108]. On the other hand, another study showed elevated RUNX2 expression in all tumor subtypes, but its highest level was found in HER2/ErbB2 positive tumors and a significant poor prognosis was associated with the ER-negative subtype of breast tumor [109].

Many cancers preferentially metastasize to bone and that is believed to be a result of osteomimicry, associated with the expression of several genes specific for bone tissue [110]. RUNX2, as a master regulator of skeletal homeostasis, and also a factor involved in carcinogenesis, seems to be a prime candidate to play this role. In fact, RUNX2 influence several genes involved in cancer cells metastasis and invasion, such as *BSP*, *MMPs* (Matrix Metalloproteinases), or *VEGF* [111,112,113].

**Figure 4:**
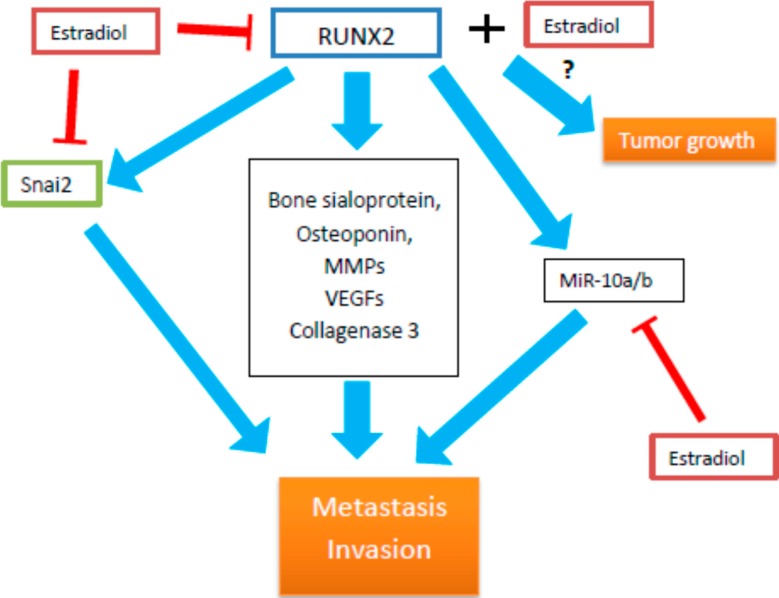
Important role of RUNX2 in estrogen-dependent breast cancer development and progression. Blue arrows indicate positive influence, while red are negative. Snai2: breast cancer-related transcription factor; MMPs: matrix metalloproteinase; VEGF: vascular endothelial growth factor.

The results of some studies suggest overlapping roles for estrogen and RUNX2 in carcinogenesis. The interplay between RUNX2 and estrogen signaling was reported in several cancers. Selective modulators of estrogen receptors were shown to induce RUNX2 expression [114]. Moreover, RUNX2 physically and functionally interacts with estrogen receptors [95]. RUNX2 appears especially important in estrogen-negative breast cancer, and its level is directly associated with a poor prognosis in this type of breast cancer [109]. It is possible that, in this case, RUNX2 and ER pathways act in an opposite way, and ER expression diminishes pro-malignant effects of RUNX2 in breast carcinogenesis. In fact, another study confirmed that ER was capable of inhibiting RUNX2 in breast cancer cells [74]. Moreover, deletions in the DNA binding domain in ER eliminated the influence of estrogen on RUNX2 activity [115]. A strong positive association was shown for RUNX2 expression and both estrogen and progesterone receptors in G2 grade breast tumors, and there was a significant difference between G2 grade and G1/G3 grades (a higher tumor grade means less differentiated cells within the tissue specimen) [108]. It was suggested that RUNX2 is expressed early during cancer progression and might be responsible for early events of tumor development [108]. Surprisingly, in that study, the subset of cancer cells showed nuclear staining for RUNX2 correlated with ER, suggesting that in those cells both RUNX2 and estrogen receptor acted synergistically, stimulating cancer development [108]. In summary, it may be concluded that, not only the expression of estrogen receptors, but also the expression of RUNX2, can differentiate types of breast cancer. An increased expression of RUNX2 and miR-10a/b in ER-negative and triple-negative breast tumors was observed. MiR-10a/b, belonging to mir-10 family of microRNA, is known to play a role in tumorigenesis and cancer progression in many cancers [116,117,118,119]. RUNX2 was shown to upregulate miR-10a/b and promote breast cancer cell migration and invasion [34]. These studies suggest opposite roles of RUNX2-dependent and ER-dependent pathways in MCF-7 breast cancer cells [104]. In this study, estradiol stimulated cancer cell colonies growth, while RUNX2 inhibited this process, acting as a tumor suppressor. Further transcriptome analysis confirmed reciprocal inhibition of RUNX2 and estrogen signaling, since the expressions of estradiol- and RUNX2-regulated genes were inversely correlated [104] (Figure 4).

#### 4.2.2. HER1 and HER2 Receptors

Some studies also suggest that other breast cancer-related factors may be involved in the association between RUNX2 and estrogen signaling. Many molecular markers for breast cancer were identified, and used in diagnostic practice, including HER1 and HER2 receptors, belonging to the epidermal growth factor receptor family [120]. Their cytoplasmic C-domain has tyrosine kinase activity capable of signal transduction into two main downstream pathways, MAPK and PI3K pathways [121]. Both, HER1 and ERs participate in the regulation of breast tumor growth and their expression is negatively coupled—an increase in HER1 is associated with a decrease in ERs and *vice versa* [122]. Breast tumors with upregulated HER2 (HER2-positive) display a more aggressive phenotype, including a higher proliferation rate, and more intense angiogenesis, invasiveness, and resistance to apoptosis [120]. HER2 overexpression occurs in 15%–20% of breast cancer patients and is correlated with a lower survival rate [123]. Estrogen, through its receptors, is capable of activating HER2 signaling. Patients with HER2- and ER-positive breast cancer are diagnosed at younger age, with tumors of higher stage and grade, and with more intensive vascular invasion [124]. It is possible that RUNX2 cooperates with HER1 and HER2 receptors in breast cancer cells and other cell types. HER1 receptor is expressed in osteoblasts and regulates both their proliferation and differentiation [125]. In breast cancer cells, high nuclear level of RUNX2 correlates with HER2 cellular status and this is associated with poor prognosis, although the worst outcome is linked with high RUNX2 and HER2-negative cases [109].

Many cells of osteoblastic lineage express significant level of HER1 [126], although its function in osteogenesis is not completely known. Studies on animals demonstrated that downregulation of HER1 in osteoblasts reduced its development and led to defects in bone formation [126]. It was shown that HER1 regulated RUNX2 transcription, acting mainly on P1-originated RUNX2 II isoform [127]. HER1 upregulated HDACs 4 and 6 and downregulated RUNX2 II isoform in MC3T3-E1 cells [127]. RUNX2 levels were reported to correlate with a number of clinico-pathological indicators, including tumor stage, histological grade, and HER2 status [109]. Since high HER2 and RUNX2 status are independent poor prognostic factors in breast cancer, both parameters together seems to be a novel and combined indicator for prognosis assessment and further studies on the cooperation of both these factors are justified.

#### 4.2.3. Cyclin D1

Cyclin D1 is a key regulator of cell cycle progression, it also stimulates development of many tumors, including breast cancer [128]. Cyclin D1 in a complex with cyclin-dependent kinases, Cdk4 and Cdk6, is involved in the regulation of G1/S transition. It phosphorylates Rb, promoting DNA synthesis [129]. Cyclin D1 was reported to be overexpressed in breast cancer, although there are some concerns whether it may serve as a universal breast cancer prognostic factor due to some contradictory data [130]. However, a correlation was shown for ER-positive breast cancers, demonstrating that cyclin D1 was strongly linked with estrogen and poor prognosis in ER-positive cases [131,132,133]. Moreover, it is known that cyclin D1 is linked with estrogen signaling and can act as a cellular ER sensor and contribute to ER activation in breast cancer cells [130,134,135]. Cyclin D1 presence is also required for the activation of many genes dependent on estrogen [136]. Estrogens, in turn, were shown to stimulate activation protein-1 and specificity protein-1, which in turn increased the expression of cyclin D1 [137]. Cyclin D1 was reported to cooperate with RUNX2, as deletions of RUNX2 were associated with a reduced expression of cyclin D1 and decreased cell proliferation and increased survival rate [138]. In breast cancer MCF-10A cell line, elevated RUNX2 was associated with upregulation of D1 [138]. However, an elevated level of cyclin D1 was shown to decrease the level of RUNX2 [139]. The exact mechanism of interaction between RUNX2 and cyclin D1 in breast cancer is not fully known, although it may have features of those in osteoblasts lineage. It was also shown that the cyclin D1-Cdk4 complex promoted RUNX2 degradation, thus modulating its transcriptional activity in osteogenic progenitor C3H10T1/2 cell line. Cyclin D1-Cdk4 complex induced phosphorylation of serine 472 residue in RUNX2 leading to its ubiquitination and proteasome-dependent degradation [139]. This effect can be considered as a mechanism of switching between proliferation and differentiation of breast cancer cells. Another study showed that RUNX2 could be a key component responsible for breast cancer metastasis to bone [128]. It was shown that RUNX2 acted through Tgfβ signaling, influencing its downstream target, PTHrP, and then cyclin D1. Cyclin D1 is an important downsteam gene of Tgfβ and PTHrP in osteogenesis, and similar a connection was observed in breast cancer [128]. Cells from bone metastatic breast cancer expressed exceptionally high level of RUNX2 [111,140] and that was associated with a high tendency of cancer cells to metastase to bone. This action of RUNX2 is associated with its influence on the activity of several downstream genes, including VEGF or matrix metalloproteinases (MMPs)-2, -9, and -13 [128,140,141]. Cyclin D1 is also a direct target for β-catenin in breast cancer cells [142]. β-catenin plays multiple roles within the cell, including involvement in cell adhesion and a number of signaling cascades, such as canonical Wnt signaling [143]. The cooperative functions of β-catenin and cyclin D1 in mammary gland development has been established, and their involvement in breast cancer development have also been shown [143]. An association between the expression of cyclin D1, β-catenin, and ER-β receptors in breast cancer cells was observed [143]. Importantly, RUNX2 expression is also under the control of β-catenin [144]. Since β-catenin is a common regulator of RUNX2 and cyclin D1, and its expression correlates with ER-β receptors in breast cancer cells, it may be considered as another important element of RUNX2-related pathways in breast cancer development.

#### 4.2.4. Matrix Metalloproteinases (MMPs)

Matrix metalloproteinases are a family of zinc-dependent endopeptidases playing a central role in the degradation of extracellular matrix (ECM). They are important in many biological processes, including embryonic development and tissue repair, but also play a role in tumor growth and metastasis [145,146]. They are useful clinical marker in breast cancer, as they are coupled with estrogen signaling in breast tumor. Moreover, some results indicate that MMPs are functionally associated with RUNX2. According to available data, many members of the MMP family appear to be potential diagnostic and prognostic markers for breast cancer, but they can also be useful in prediction of tumor recurrence, metastasis, and therapy response [147]. As an example, MMP-9 is elevated in breast cancer patients, including tumor cells; additionally, serum, plasma, and urine may be also useful for early cancer diagnosis and metastasis prediction, and MMP-13 correlates with aggressiveness of the tumor and poor prognosis in HER2-positive tumors. MMP-1 is upregulated in precancerous cells and the elevated expression of MMP-14 correlates with a shorter overall survival [147,148,149,150]. In general, MMP-1, -9, -12, -14, and -15 are significantly associated with poor prognosis in breast cancer [151]. The relationship between MMPs and the estrogen pathway in breast cancer is not clear yet. It was reported that MMPs-1 and -13 were stimulated by both ERα and ERβ receptors, but these effects depended on the presence or absence of estrogen [152]. In addition, it seems that different MMPs are differentially sensitive to the influence of ERα and ERβ isoforms [152,153,154]. There are also a number of reports showing the cooperation between RUNX2 and MMP family members, including in both normal and cancerous cells. MMPs, similarly to RUNX2, are important for bone metabolism [155]. MMP-9 and MMP-13 are under the direct control of RUNX2 in a number of cell types, including breast cancer cells, and this was associated with invasive behavior of cancer cells [111,156,157,158]. MMP-1 and MMP-2 could be also under the control of RUNX2 [157,159]. In turn, MMP-1 overexpression led to a decrease in RUNX2 level, while MMP-1 knockdown caused further decrease of RUNX2, also demonstrating that MMPs may modulate RUNX2 [160]. These data show that the relationship between the MMP family and RUNX2 is complex and still unclear, although it seems that the role of the interaction between RUNX and MMPs in estrogen-dependent breast cancer might be a promising direction for further, clinically-relevant research.

## 5. Future Perspectives

The involvement of estrogen signaling in breast cancer development may be considered on many levels, and its mechanism of action can be complex and tumor type-specific. In contrast to breast cancer, lung and digestive tissues seem to be under the protective effect of estrogen and many reports suggest such an effect in colorectal cancer [6,161,162].

The interaction between RUNX2- and estrogen-dependent pathways can contribute to the mechanisms involved in cancer transformation. RUNX2 also play a role in several pro-malignant pathways, and this line of research is currently gaining emerging interest. Although the involvement of RUNX2 in estrogen-dependent cancers has been suggested in many studies, mechanisms of its action in such malignances is still unclear. Especially, little is known about the combined effect of both estrogen- and RUNX2-dependent pathways in these cancers, although available reports suggests an essential role of this cooperation. This seems to be especially important since estrogen-dependent cancers are among the most frequent and are characterized by high mortality. Although available data are limited and contradictory in many cases, the involvement of RUNX2 in breast cancer can lead to a deeper understanding of the molecular biology of this malignancy, and provide information useful for its diagnosis and therapy.
